# Socioeconomic Position and Picky Eating Behavior Predict Disparate Weight Trajectories in Infancy

**DOI:** 10.3389/fendo.2018.00528

**Published:** 2018-09-18

**Authors:** Amy T. Galloway, Paul Watson, Suzanne Pitama, Claire V. Farrow

**Affiliations:** ^1^Department of Psychology, Appalachian State University, Boone, NC, United States; ^2^Ara Institute of Canterbury, Christchurch, New Zealand; ^3^Royal New Zealand Plunket Trust, Wellington, New Zealand; ^4^Māori/Indigenous Health Institute, Otago University, Christchurch, New Zealand; ^5^Department of Psychology, School of Life & Health Sciences, Aston University, Birmingham, United Kingdom

**Keywords:** picky eating behavior, pressure to eat, socioeconomic position, infant weight trajectory, appetitive phenotype

## Abstract

Infant weight gain has long-term implications for the establishment of overall health. We examined whether socioeconomic position (SEP), the use of pressure as a feeding practice, and picky eating relate to changes infant in weight-for-length (WFL). A modified developmental design was used to examine whether current levels of child picky eating, parental use of pressure, and SEP were associated with changes in WFL during infancy. Health providers distributed survey packets during routine well-child visits made in the homes of families with young children in New Zealand (*n* = 193). Primary caregivers of young children provided their child's current level of picky eating, their use of pressure, and their SEP. They also reported their child's professionally-measured WFL from birth, 8, 15, and 21 months of age. A multi-level modeling analysis yielded an interaction between SEP and picky eating in predicting infant weight change over time. Children who had a low SEP and were not picky eaters were on the highest WFL trajectory and children who had a low SEP and were picky eaters were lowest on the WFL trajectory. A main effect revealed that higher levels of parental pressure predicted lower WFL in infants at each age, but did not interact with SEP or picky eating. Findings from this study indicate that the combination of eating behavior and SEP are associated with differential infant growth patterns. These results suggest that eating behavior and SEP should be included in the development of interventions designed to achieve healthy weight during childhood.

## Introduction

The period representing the transition from milk feeding to independent eating has been scarcely studied with regard to infant eating behavior, growth, and parental feeding practices, yet the development of infant weight status has implications for the long-term physical, cognitive, and socio-emotional well-being across the globe (1). Understanding how eating behavior develops during the first 1,000 days of life is useful for designing interventions for healthy eating patterns (2). New Zealand (NZ) is a high-income country where children living in socioeconomically deprived communities are three times as likely to be obese than their less deprived counterparts (3). Although several studies have corroborated the inverse relationship between socioeconomic position (SEP) and weight status, the relationship is not well understood (4). For instance, although some studies suggest that low SEP predicts growth faltering in infants, others dispute this finding (5, 6). Further, low-SEP infants may not demonstrate an inverse relationship between SEP and weight as documented in older children (4, 7). Some research suggests that both slow growth and rapid growth during infancy may lead to the development of overweight in later childhood (2). Although food security is likely to be a factor that determines the impact of SEP on child weight, there are several other potential factors that moderate the relationship between SEP and child weight gain or loss. Two such factors that we explore in the present study are eating behavior, particularly picky eating, and the parental feeding practice of pressuring a child to eat.

Picky, or fussy eating can be described as the rejection of a number of foods that results in low dietary variety and low food intake in general (8). However, the definition and measurement of this eating behavior has been inconsistent in the literature (9). Picky eating behavior is often conceptualized as a normal and transient behavior in children, in which only the most extreme cases, indicating nutritional inadequacy, represent disordered eating, and require intervention (10). Research indicates that picky eaters consume lower quantities of some micronutrients and fiber (11, 12) and are more likely to be constipated (13). In older children, picky eating has also been shown to be associated with the development of psychopathology and poor family functioning (14). Picky eating behavior has been linked to both underweight (15, 16) and overweight (17–19) in various studies, and a recent systematic review corroborates these conflicting findings, indicating a problem with inconsistent operational definitions (20). A recent longitudinal analysis revealed no relationship between picky eating and weight gain over time (21). During infancy, picky eating has been shown to be inversely related to weight status and reduced food intake and variety (22). Having an infant who is a picky eater is stressful for parents and is likely to be a common reason that caregivers consult health professionals and pressure or force their child to eat (15). In a recent longitudinal study, children were more likely to be picky eaters at age six if their parents were rated as less “sensitive” during observed interactions with their child 2 years earlier (23).

Pressure to increase the quantity or quality of food a child eats has also been hypothesized to influence child weight status because its use may desensitize children to their internal cues of satiety, thereby making them less able to self-regulate their intake of food (24–26). Pressure to eat often is associated with smaller size in infants and preschoolers, and lower food intake in general (15, 16, 27), but one recent longitudinal analyses reported no long-term effects of pressure (28). The relationship between pressure to eat and weight in children is thought to be bidirectional and dependent on context. Some researchers have suggested that parents may respond to lower child weight by pressuring the child to eat, which then has the counterproductive effect of disrupting self-regulation and intuitive eating over time (25). However, some types of pressure to eat have been shown to predict higher food intake, higher weight status, or greater eating in the absence of hunger (24, 29–31), possibly because parents react to perceived lower weight by pressuring the intake of energy-dense foods (24). In one recent longitudinal study researchers reported there was no indication that pressure at an earlier age predicted higher levels of picky eating or growth over a two-year period (28). The exact effect of pressure to eat is not well understood and it is likely that this feeding practice has different outcomes depending on the quality of the interactions with the child and the context in which they occur (29–31). Pressure to eat may be immediately effective in increasing intake, but may undermine self-regulated eating in the future (32). Moreover, forceful feeding is unlikely to result in food liking, but gentle prompting to eat may be effective at encouraging children to taste novel foods (33, 34).

Positive encouragement to eat may be particularly relevant for families facing low SEP where food supplies are limited and food insecurity is a concern. Considering an infant's socioeconomic position as a contextual variable may be important for understanding the bi-directional relationships between child eating behavior, parental feeding practices, and infant growth (15, 35). However, most research in this area comes from studies in the US or UK with participants from middle or upper-class families that include older children. There is a growing body of literature suggesting that the caregiver-child behavioral interactions should be included in the development of effective interventions for infants (27, 36, 37). In this study we explored whether SEP, picky eating behavior, and parental feeding practices influence weight change over time. We hypothesized that low SEP would predict both low and high WFL trajectories, and that parental pressure to eat and the level of picky eating would moderate these relationships.

## Materials and methods

### Participants

Well Child Health Providers, including nurses and social workers from the Tamariki Ora Programme in Canterbury, NZ, recruited caregivers for this study. Health providers distributed invitational letters and survey packs to caregivers with whom they visited routinely during well-child visits in family homes or community health clinics. Any caregiver enrolled with a well-child health provider with at least one child between the ages of 1–5 years was eligible to participate in the study. The providers invited caregivers to participate in the study during a single routine visit. Participants were given the option to complete the survey over the telephone, in person with a researcher, or individually and then returned the survey via post. All participants used this latter method. This study was carried out with the approval of, and in accordance with, the recommendations of the institutional review board at the NZ Ministry of Health, Appalachian State University (USA), Christchurch Polytechnic Institute (now Ara Institute of Canterbury - NZ), as well as from participating organizations, including the Otautahi Māori Women's Welfare League, the Pacific Trust Canterbury, and Royal New Zealand Plunket Trust. Participants provided consent by choosing to complete the survey. The surveys remained anonymous when participants mailed them back to the researchers.

Aligned with the New Zealand Ministry of Health guidelines, parents self-identified the ethnicity of their child, by completing the standard ethnicity question. This question allows for the inclusion of multiple ethnicities. Analysis of ethnicity, in line with guidelines, is usually reported in two ways. First, in line with the obligations under The Treaty of Waitangi (the founding document of New Zealand) between Māori tribal groups and the Crown, reported outcomes are presented as a comparison between Māori and Non-Māori (38). This approach allows Māori to monitor the Crown's responsiveness to equitable outcomes in a range of areas including the determinants of health. The Non-Māori group consists of all those who do not identify any of their ethnicities as Māori. The second reporting format most commonly used in the health and disability sector is prioritization of ethnicities, where respondents who enter more than one ethnicity are assigned to a single ethnic group, for the purposes of analysis. The priority order is Māori, Pacific Peoples, Asian, Middle Eastern/Latin American/African (MELAA), Other Ethnicity, and European. The ethnicity of the study population was very similar to the Canterbury Regional population aged 0–4 years from which it was drawn; although the ethnicity of the Canterbury Region population is significantly different from the total New Zealand population aged 0–4 years (39).

### Procedure

Approximately 950 survey packets were available to be distributed during well-child visits and 193 surveys (20%) were returned. Given that health-care providers volunteered to distribute the survey packets for this study, we were unable to assess how many potential participants received a survey. Three organizations were selected to invite families to participate during routine health visits: Royal New Zealand Plunket Trust, Otautahi Māori Women's Welfare League, and Pacific Trust Canterbury. These organizations were selected because they serve the majority of children in the Canterbury area and to ensure Māori and Pacific peoples who are often under-represented in such studies were adequately represented in the study sample. When caregivers completed they surveys, they provided both current (i.e., demographic, child eating behavior, child feeding practices) and past information (i.e., child lengths/heights and weights beginning at birth) about themselves and their young child. In addition, the age range of children when the surveys were completed were between 1 and 3 years of age.

### Measures

#### Background and anthropometric measures

Caregivers provided background information about themselves (ethnicity, weight status) and their children (sex, self-identified ethnicity, health history). Ethnicity was recorded using the NZ statistical guidelines (40). In New Zealand, it is customary for a nurse to visit all infants in their home for wellness checkups after birth and then at 8, 15, and 21 months of age. During this visit, the nurse measures the infant and records height and weight information in a booklet kept by parents called the Tamariki Ora: Well Child Health Book. Height, weight, age, and gender data were used to calculate WFL scores for children using World Health Organization growth charts (41). Parents were asked to enter the weight and height information on the survey that had been previously recorded in the booklet.

#### New Zealand individual deprivation (NZiDep)

The NZiDep is a non-occupational index, to measure SEP among NZ citizens (42). NZiDep contains eight items that measures increasing levels of deprivation using a yes or no response format. The NZiDep scores are then assigned to one of 5 deprivation groups ranging from 1 (no deprivation factors) to 5 (more than 5 deprivation factors), with high scores indicating more severe deprivation. Examples of items include, “In the last 12 months have you personally made use of special food grants or food banks because you did not have enough money for food?” and “In the last 12 months have you personally gone without fresh fruit and vegetables, often, so that you could pay for other things you needed?” NZiDep has good construct validity, criterion validity, and internal reliability, α = 0.81 (42). Its strengths include relevance to the current New Zealand context, acceptability across ethnic groups, and three of the eight questions relate closely to items in the food security survey used as part of the children's nutrition survey (3). In the current sample, α = 0.71.

#### Child feeding questionnaire (CFQ)

The CFQ assesses parents' perception of the feeding practices used with their children (43). Four relevant subscales were used for this study including: concern about child weight (4 items), pressure to eat (4 items—the degree to which parents report encouraging their child to eat), restriction of food (8 items—parental behaviors that restrict children from eating certain foods), and monitoring (3 items—the degree to which parents report keeping track of the foods their child eats). The CFQ has good internal consistency and has been used in the US, UK, and Australia. Minor word alterations were used to make the questionnaire comprehensible for a New Zealand sample. In the current sample, all subscales had acceptable internal reliability, including pressure to eat (α = 0.71).

#### Children eating behavior questionnaire (CEBQ)

The CEBQ measures parents' perceptions of their child's eating behavior using 35 items comprising 8 subscales including: food responsiveness, emotional over-eating, enjoyment of food, desire to drink, satiety responsiveness, slowness in eating, emotional undereating, and food fussiness (44). Parents respond whether they believe their child demonstrates the behavior described in each item. Response options range from 1 (never) to 5 (always). The CEBQ has acceptable internally reliability (α = 0.72–0.91) and test-retest reliability (44, 45). Carnell & Wardle (45) showed that three of the CEBQ subscales (Satiety Responsiveness, Food Responsiveness, and Enjoyment of Food) have good external validity because they predict obesigenic behavioral measures in children. It also has been shown to have good external validity for four subscales that have been tested (45–47). In the current sample, all subscales had acceptable internal reliability (α = 0.73–0.90), with the exception of the emotional over-eating subscale (α = 0.65).

Tharner et al. examined the complexity of picky eating behavior using a latent profile analysis (LPA) with the CEBQ subscales. Instead of using the single “fussy” subscale, individual participants were assigned a profile comprised of their scores on both food avoidant and food approach subscales (15). We conducted an LPA using z-scores on the five CEBQ subscales to develop eating behavior profiles. To determine the best fitting model, we referred to several fit indices and assessed the meaningfulness of the profile solution and the size of each class. We settled on a three-profile solution which classified children as Picky eaters, Moderate eaters, or Joyful eaters because the bootstrap likelihood ratio test suggested that it was significantly better than a two-profile solution and that there was a non-significant improvement with four profiles. In addition, the adjusted BIC index diminished slightly with the four-profile solution and the three-profile solution was more parsimonious and theoretically meaningful. In this study, we use the terms, “picky”, “moderate”, and “joyful” to correspond with the use of the CEBQ measurement tool and the Tharner et al. (15) analytic approach.

### Statistical analyses

Descriptive statistics were first computed on the demographic variables. We then examined whether there were significant differences in child eating behavior, parental feeding practices, or socioeconomic position in terms of child ethnicity (Māori compared to non-Māori). We next ran a series of Pearson correlations to examine relations among the primary variables of interest. Tharner et al. (15) developed a method of examining the complexity of picky eating behavior by assessing a profile consisting of a combination of scores on subscales of the CEBQ. We replicated this statistical technique, such that instead of using the single “fussy” subscale of the CEBQ, individual participants were assigned a profile comprised of their scores on both avoidant and approach subscales. Following the latent profile analysis, we used the probability of having a picky eater profile as a predictor variable in a multi-level modeling (MLM) analysis. We used multi-level modeling (MLM) to test that hypothesis that SEP, picky eating, and parental pressure to eat would interact to predict child weight change over time. Given that we did not have complete WFL data for all the participants, we chose MLM because it enabled us to examine change over time and it handles missing data without needing to exclude participants (48).

## Results

We examined 193 parent-child dyads. From the sample of caregivers, 178 participants were mothers, 1 was an adoptive or foster mother, 3 participants were fathers, and 1 did not disclose their relationship status with the child. Caregivers indicated on the survey the ethnic group to which their child belonged. The ethnic groups were not exclusive in that participants could select more than 1 group. Caregivers had the option of choosing identifications for their children using one ethnic group (81%) or two or more ethnic groups (36%). Following research protocols set out by the Treaty of Waitangi (38), we categorized participants as Māori (*n* = 31) or non-Māori (*n* = 160) ethnicity. If caregivers identified their children as Māori and one or more other ethnicities, we classified the children as “Māori”, using the prioritization protocol. Based on the identities marked by caregivers, 142 (74%) of children were New Zealand European and 31 (16%) classified as Māori, indicating that this sample is representative of the Canterbury region of NZ (39). Two participants chose not to disclose this information. Other ethnicities identified by mothers included 3 Samoan, 2 Tongan, 4 Chinese, 1 Indian, 1 African, 1, Latin American, 1 Other European, 2 Other Asian. There were 3 children identified as “Other Ethnicity” (i.e., United States).

Table 1 provides an ethnic comparison of socioeconomic deprivation. Caregivers reported that 47.0% of the children were female. Infant had a mean birth weight of 3.46 kgs (*SD* = 0.70), girls weighing a mean of 3.32 kgs (*SD* = 0.80) and boys weighing 3.57 kgs (*SD* = 0.59) at birth. Children were a mean age of 30 months (*SD* = 12.81) when their caregiver completed the survey. The mean parent age was 33 years (*SD* = 5.32) and the mean parent BMI was 25 (*SD* = 5.41), suggesting borderline overweight. Additional descriptive statistics for the sample are provided in Table 2. There were no ethnic differences between Māori and non-Māori participants in infant WFL scores at 8, 15, and 21 months, Table 3. Parent BMI and parent age were not related to infant WFL at 8, 15, or 21 months. These results did not change when the Pacific families (*n* = 5) were removed from the non-Māori category in the analysis.

**Table 1:** Socioeconomic position (SEP) scores by ethnicity (percent).

**SEP value**	**Non-Māori *n* = 160**	**Māori *n* = 31**	**Total *n* = 193**
1	53.1	29.0	34.3
2	21.3	29.0	15.9
3	11.3	16.1	8.3
4	12.5	12.9	8.7
5	1.9	12.9	2.5

*Higher SEP scores indicate more severe socioeconomic deprivation. Two participants did not provide ethnicity identification. Non-Māori ethnic identifications include European, Pacific, Asian, and Other. The results did not change when Pacific families (n = 5) were excluded from the Non-Māori category*.

**Table 2:** Difference scores for Māori and Non-Māori families on child feeding, child eating, and socio-economic status.

**Variables**	**Full sample *n* = 193**	**Non-Māori *n* = 160**	**Māori *n* = 31**	**Difference (*t*)**
Characteristics				
Child gender (% female)	47	45	55	
Parent age	33.17 (5.32)	33.90 (4.80)	29.35 (6.31)	4.57^***^
Parent BMI	25.04 (5.41)	24.72 (5.38)	26.77 (5.30)	−1.74
Socioeconomic deprivation	1.98 (1.20)	1.89 (1.14)	2.52 (1.39)	−2.70^**^
Child feeding practices				
Monitoring	4.47 (0.75)	4.51 (0.77)	4.31 (0.66)	1.27
Concern about child weight	2.06 (1.14)	1.99 (1.12)	2.49 (1.20)	−2.26^**^
Pressure to eat	2.63 (1.00)	2.60 (1.01)	2.77 (0.93)	−0.90
Restriction	3.32 (0.85)	3.27 (0.88)	3.60 (0.64)	−2.41^*^
Child eating behavior profiles				
Joyful eater	0.28 (0.38)	0.27 (0.38)	0.30 (0.38)	−0.32
Moderate eater	0.52 (0.41)	0.50 (0.41)	0.63 (0.40)	−1.70
Picky eater	0.20 (0.38)	0.23 (0.39)	0.07 (0.25)	2.19^*^

*Values reported as Mean (SD) except for gender. Higher scores indicate higher level of characteristic or behavior. Two participants did not provide ethnicity identification. Non-Māori ethnic identifications include European, Pacifica, Asian, and Other. The results did not change when (n = 5) Pacific families were excluded from the Non-Māori category*.

* p < 0.05;

** p < 0.01;

*** *p < 0.001*.

**Table 3:** Descriptive values for weight-for-length Z scores over time for full sample and for samples dichotomized by ethnic classifications.

**Weight-for-length Z scores**	**Full sample *n* = 193**	**Non-Māori *n* = 160**	**Māori *n* = 31**	**Difference (*t*)**
Birth	*n* = 163 −1.33 (1.58)	*n* = 136 −1.31 (1.56)	*n* = 25 −1.27 (1.63)	−0.11, *ns*
8 months	*n* = 140 0.25 (1.01)	*n* = 136 0.21 (0.99)	*n* = 19.61 (1.09)	−1.63, *ns*
15 months	*n* = 157 0.64 (0.86)	*n* = 124 0.63 (0.86)	*n* = 14 0.84 (0.76)	−0.87, *ns*
21 months	*n* = 107 0.73 (0.95)	*n* = 94 0.70 (0.95)	*n* = 11 1.26 (0.56)	−1.91, *ns*

*Values reported as Mean (SD). Non-Māori ethnic identifications include European, Pacifica, Asian, and Other. The results did not change when Pacific families (n = 5) were excluded from the non-Māori category, nor when Non-parametric statistics were used to analyze differences*.

Following Tharner's (15) approach of developing eating behavior profiles, we conducted a latent profile analysis in Mplus using z-scores on the five CEBQ subscales. To determine the best fitting model, we referred to the AIC index, the BIC index adjusted for sample size, relative entropy, the Lo-Mendell-Rubin Likelihood Ratio Test, and the Bootstrap Likelihood ratio test. In addition to fit indices, we also looked to the meaningfulness of the profile solution and the size of each class. We settled on a three-profile solution for the following reasons. First, the bootstrap likelihood ratio test suggested that it was significantly better than a two-profile solution, while the same test suggested nonsignificant improvement with four profiles. Second, adjusted BIC got only slightly smaller with the four-profile solution. Finally, the three-profile solution (Picky, Moderate, and Joyful eaters) was more parsimonious and theoretically meaningful.

Table 4 includes associations between child feeding practices and the eating behavior profiles. Parental pressure to eat was positively associated with the food picky eater profile and negatively associated with the joyful eater profile. Table 5 shows the correlations between predictor variables and the outcome measures of WFL scores through infancy. The results indicated no relationship between SEP and WFL scores over time. Parental use of pressure to eat as a feeding practice was consistently related to lower WFL. There was a strong pattern of picky-type eating behaviors and the picky eater profile related to lower weight status over time. Less consistently, the joyful eater profile of behaviors was related to higher weight status.

**Table 4:** Pearson correlations between child feeding practices and child eating behavior (*n* = 193).

**Variables**	**Concern about child weight**	**Monitoring**	**Restriction**	**Pressure to eat**
Child eating behavior profiles Picky eater Moderate eater Joyful eater	−0.212^**^ 0.081 0.122	0.031 −0.040 0.014	0.029 0.022 −0.052	0.266^**^ 0.084 −0.175^*^

* p < 0.05;

** *p < 0.01*.

**Table 5:** Pearson correlations among socioeconomic position, feeding practices, eating behaviors, and weight-for-length Z scores.

**Variables**	**Socioeconomic position *n* = 193**	**Birth WFL *n* = 163**	**8-month WFL *n* = 157**	**15-month WFL *n* = 140**	**21-month WFL *n* = 107**
Socioeconomic position (completed once during home visit)		−0.054	−0.058	−0.005	−0.166
Parental feeding practices and concerns (completed once during home visit)					
Concern about child weight Monitoring Restriction Pressure to eat	0.192^**^ 0.008 0.029 0.176^*^	−0.042 −0.152 −0.026 −0.028	0.144 −0.015 −0.044 −0.288^**^	0.127 0.124 −0.011 −0.205^*^	0.167 −0.068 −0.124 −0.329^**^
Child eating behavior profile (completed once during home visit)					
Picky eater Moderate eater Joyful eater	0.057 −0.055 0.003	−0.141 0.119 0.011	−0.273^**^ 0.116 0.152	−0.320^**^ 0.155 0.146	−0.418^**^ 0.202^*^ 0.235^*^

* p < 0.05;

** *p < 0.01*.

The fully unconditional model indicated that 37.3% of the total variance in WFL was due to individual change over time (within-subjects variance), σ^2^ = 0.35, *z* = 10.87, *p* < 0.0001, and 62.7% of the total variance in WFL was due to between-subject differences, τ_00_ = 0.59, *z* = 7.00, *p* < 0.0001. The next set of analyses examined the linear effect of time (e.g., age in months) on WFL. The first model tested constrained the slope to be the same across participants [e.g., a One-Way ANCOVA with Random Effects Model; (48)]. This analysis indicated that age in months was associated with a higher WFL, γ_10_ = 0.04, t = 6.18, *p* < 0.0001, accounting for 12.5% of the within-subjects variance. Allowing the slopes to be free to vary across people [e.g., a Random Coefficients Regression Model; (48)] resulted in a better model fit, with age in months, γ_10_ = 0.04, *t* = 5.84, *p* < 0.0001, accounting for 36% of the within-subjects variance. Because of this, slopes were allowed to vary in the subsequent analysis.

**Figure 1:**
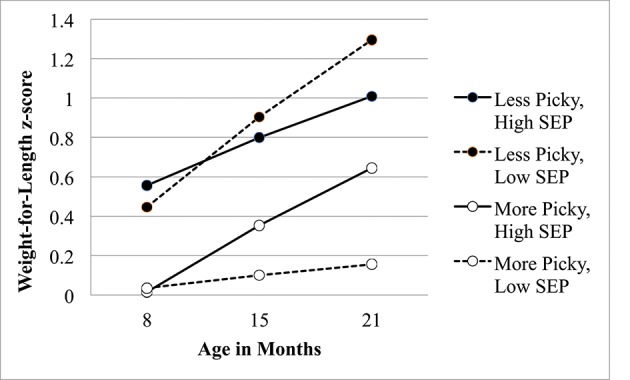
Interaction of picky eating behavior and socioeconomic position on weight-for-length z scores during infancy. SEP, Socioeconomic Position; Higher SEP scores indicate more severe socioeconomic deprivation.

Finally, the effects of SEP, pressure to eat, and picky eating on WFL change, along with interactions among these variables, were tested with a single Intercepts and Slopes as Outcomes model (48). For this model, only two effects were significant: the main effect of pressure to eat, γ = −0.26, *z* = −2.22, *p* = 0.03, indicating that greater pressure to eat was associated with lower average WFL scores at each time point. In addition, the cross-level interaction between SEP, picky eating, and child age was a significant predictor of child WFL z-scores over time, γ = −0.04, *z* = −2.12, *p* = 0.04, indicating that an infant's level of picky eating moderates the relationship between SEP and child weight gain. The relationship between WFL and pressure did not change over time, as indicated by a lack of cross-level interaction with age, γ = 0.008, *z* = 1.23, *p* = 0.22. The cross-level interaction between SEP, picky eating, and age is depicted in Figure 1.

## Discussion

This study shows that among low-SEP infants living in a high-income country, those reported to be most picky had the lowest WFL over time and those reported to be the least picky had the highest WFL over time. Parental pressure to eat was associated with lower child WFL but pressure to eat did not moderate the impact of SEP on weight change over time. Picky eating behavior significantly moderated the impact of SEP on WFL throughout infancy. These findings support previous research documenting relationships among SEP, eating behavior, child feeding practices, and WFL scores. However, to our knowledge this is the first study focused on infancy demonstrating that a specific aspect of child eating behavior moderates the effects of SEP resulting in growth trajectories situated on opposite ends of the weight spectrum.

Previous studies indicated that low-SEP mothers are more likely to report problems with persistent picky eating and to rate their child as having a responsive or approaching eating style (15), linking low SEP to both underweight and overweight during childhood (5, 49). The factors that determine the weight trajectories of children from low-SEP families are not well understood (24, 35, 49). Recent work has shown that another type of eating behavior, satiety responsiveness, is linked to a genetic predisposition for weight status and that child characteristics may be an important predictor of weight faltering and weight gain (6, 50, 51). The current results corroborate a tenet of Behavioral Susceptibility Theory that some appetitive processes, such as cue responsivity, may be predictive of weight gain (52). From an intervention perspective, it is helpful to know that although these behaviors are likely to have a biological basis, there is ample evidence that they are modifiable behavioral phenotypes (53).

Why do SEP and levels of picky eating interact to influence WFL scores over time? Recent research suggests a myriad of reasons that SEP may put infants at risk for weight disparities. They include the quality of breastmilk and the contextual factors associated with early feeding or the availability of nutrient-dense complementary foods (1). Caregiver feeding practices are also implicated in the process. Caregivers may use pressure when infants are lean and use more restriction when food is abundant (35). Low-income mothers have been shown to have more concern about infant hunger and are more likely to feed their infants on a schedule (20, 54). In this sample, low-SEP caregivers were more likely to be younger. The relationship between picky eating and age of the mother has been shown in other studies [i.e., (15)] as well as this study. It is possible that younger caregivers with less experience might be less able to respond appropriately to infants with extremely high or low picky eating behavior and they may have had fewer opportunities to be exposed to nutrition education (55). Another plausible explanation for these findings is that the relationship between picky eating, SEP, and WFL is due to another factor such as health status of the infant. It is also possible that some other factor related to food insecurity interacts with individual differences in appetitive responsiveness.

This study is unique in its focus on a sample representing diverse socioeconomic positions in a country that is not typically represented in this field of research. Another strength of the study includes the use of infant anthropomorphic measurements recorded by medical professionals at several points during infancy to explore weight trajectories over time, building on previous studies that focus on weight change over just 2 periods of time (16). In addition, we used a validated measure of non-occupational socioeconomic deprivation that was developed to be culturally relevant in New Zealand and we used Latent Profile Analysis to develop a comprehensive measure of picky eating (15). However, this study is not without its limitations. The return rate of the surveys was relatively low; health providers distributed the survey packets and it is not known how many were actually received by potential participants or whether responders differed from non-responders. While anthropometric data were measured by a health professional, it should be noted that parents transcribed the measurements from their child's medical record. Given that the WFL of children and parents in this study were lower than what is typically seen in NZ, it is possible that there may be selection bias in the families that were recruited by healthcare workers or in the families that chose to participate. Few WFL scores in this sample were clinically over- or underweight, so caution should be used when interpreting these findings. Finally, although we were able to assess weight gain trajectories from birth to 2 years, the predictor variables were assessed at the time the surveys were distributed so this is not a truly prospective study.

These results suggest that the combination of particular appetitive phenotypes and factors in the home environment may have a powerful influence on the establishment of infant weight. The findings imply that knowing the relationship between SEP and children's eating behavior could be crucial for developing interventions aimed at establishing healthy infant growth. This may be particularly relevant for low- and middle-income countries, where the double burden of child underweight and overweight is particularly challenging. The current findings suggest that researchers should consider the effect that socioeconomic position has both ends of the weight spectrum, especially in light of evidence that sugary-sweetened beverages can be significant source of calories for children of all weights and therefore potentially masking what might otherwise be very low weight (56). Additional work is also needed to understand parental use of pressure and its potential for both positive and negative influences on the development of infant eating behavior.

## Data availability

The datasets for this manuscript are not publicly available because participants did not provide consent to have their data released.

## Author contributions

All authors contributed to the conceptualization and design of the study. AG, PW, SP coordinated and supervised data collection. PW and AG supervised data entry and data organization. AG and CF analyzed the data and all authors interpreted the results. AG drafted the initial paper and all authors revised the manuscript and approved the final manuscript as submitted.

### Conflict of interest statement

The authors declare that the research was conducted in the absence of any commercial or financial relationships that could be construed as a potential conflict of interest.

## Abbreviations

SEP: Socioeconomic position
WFL: weight-for-length
CFQ: Child Feeding Questionnaire
CEBQ: Child Eating Behavior Questionnaire
NZiDep: New Zealand Individual Deprivation.
